# From ethics of care to psychology of care: reconnecting ethics of care to contemporary moral psychology

**DOI:** 10.3389/fpsyg.2014.01135

**Published:** 2014-10-17

**Authors:** Aner Govrin

**Affiliations:** The Program for Hermeneutics and Cultural Studies, Department for Interdisciplinary Studies, Bar-Ilan UniversityRamat Gan, Israel

**Keywords:** ethics of care, attachment theory, moral development, infant research, moral psychology, mind perception

## Abstract

Moral psychology once regarded ethics of care as a promising theory. However, there is evidence to suggest that nowadays moral psychology completely ignores ethics of care’s various insights. Moreover, ethics of care’s core concepts – compassion, dependence, and the importance of early relations to moral development– are no longer considered to be relevant to the development of new theories in the field. In this paper, I will firstly discuss some of the reasons which, over recent years, have contributed to the marginalization of the role of ethics of care in moral psychology. Next, I will show that ethics of care’s most promising idea centered on the care given to an infant and the importance of that care to the development of moral thinking. In this context, I will be describing the implications of John Bowlby’s attachment theories, infant research, findings in moral psychology and neuroscience. I will argue that ethics of care needs to be radically re-thought and replaced by a psychology of care, an attachment approach to moral judgment, which would establish the centrality of the caregiver’s role in moral development. The philosophical implications of this approach to the understanding of the “rationalists” and “intuitionists” debate about the true nature of moral judgment is also discussed.

Ethics of care is in a puzzling situation. It is dying as a moral psychology but prospering as an ethical theory. It is very influential in disciplines such as philosophy (Slote, 2006), nursing (Bowden, 1995), social work (Bolmsjö et al., 2006), and education (Noddings, 1984). And yet, none of its core ideas impact on contemporary moral psychology. Considering that ethics of care, shaped by psychologist Carol Gilligan, was originally developed as a sub-discipline of moral psychology, its lack of influence in that particular area requires further enquiry.

Ethics of care is well recognized as a normative ethical theory – a theory about what makes actions right or wrong (Allmark, 1995). It occupies a prominent place among a cluster of normative ethical theories advanced by feminists in the second half of the 20th century. It was successful in establishing a significant alternative to Kant’s moral philosophy, utilitarianism and virtue theory (Slote, 2006). By placing the emphasis on the way in which the work and practice of care originates in the private realm, it distinguishes itself from the nurturing of the character of the Man of Virtue, or the Man of Reason’s exercise of universal rationality in the public realm (Held, 2006).

It can be assumed that a theory will mostly be employed in the field in which it resolves a conceptual or empirical problem with which that particular discipline is preoccupied. (Laudan, 1977). Nowadays, care ethics is considered to be a key concept in ethical debates linked to routine work in social work and nursing – the allocation of resources, the preference given to certain populations or patients, and the way in which a patient or any other individual in need should be treated. That particular perspective has led to the development of important insights into the moral values involved in the caring practices applied to family relations, relations between friends, and individual care giving.

Ethics of care gave to the world a way of thinking which shed new light on these issues and provided possible solutions. As a result, today the terms “caring,” “nursing,” “social work,” and “ethics of care” are inextricably linked.

In the making of moral decisions about routine issues related to care, ethics of care takes into account considerations that traditional moral theories did not recognize as legitimate. It differs from the traditional Kantian formulation according to which welfare policy has to be set according to universal laws of justice, as well as from the idea that cold rationality is more important than emotion in resolving a moral dilemma.

However, ethics of care’s influence on contemporary moral psychology is negligible. Its diminished contemporary status is in marked contrast to the promising start made during the 1980s when care ethics was the first theory to challenge Lawrence Kohlberg’s moral psychology which had dominated the field for many years. Gilligan’s (1982) book, *In a Different Voice*, has been widely read and is one of the most influential works in the feminist discourse. It was acclaimed by feminist historians and philosophers, some of whom appeared to be trying to integrate its findings and suggestions into their own scholarship (Kerber, 1986). But ethics of care has had little impact on contemporary moral psychology and is nowadays hardly discussed or mentioned within that discipline. The key theories of contemporary moral psychology include: Universal Moral Grammar (Hauser, 2006; Mikhail, 2007); Moral Foundation Theory (Haidt, 2007; Graham and Haidt, 2010); Relation Regulation Theory (Rai and Fiske, 2011); The character view of morality – how we tend not to judge specific acts but rather the agents who perform them (Alicke, 2000; Pizarro and Tannenbaum, 2012). Finally, there is the theory suggesting that moral judgments are driven by two core factors—affective reactions and cognitive processes (Nichols, 2002; Greene, 2007). None of these theories place the key concepts of ethics of care – compassion, concern, relations, and dependence – at the center of their moral thinking. Nor do they refer to ethics of care as a source of influence. Even theories that have much to say about emotions being central to moral judgment do not see in ethics of care a possible source of inspiration and make no reference to it (Nichols, 2002).

As a school of thought ethics of care is not a participant in the essential and fascinating discourse on the nature of morality which some psychologists and philosophers are engaged in. Other that Carol Gilligan, the leading figures identified with ethics of care are drawn from the fields of education, philosophy, and political science, not psychology. What was once considered as being almost the only alternative to Kohlberg’s theory, is not mentioned in the important primary books on morality such as *The Moral Psychology Handbook* (2010); *Moral Psychology : Historical and Contemporary Readings* (2010); or *Moral Psychology : A Contemporary Introduction.* The same can be said about the entry for “moral psychology” in The Encyclopedia of Philosophy published by Stanford University. In all, ethics of care has been pushed into the important but limited niche of feminist ethics.

Indeed care ethics, and feminist ethics are often treated as synonymous. According to the APA PsycNet, in the 20 years between 1992 and 2012, 45 articles were published in which ethics of care was a central topic and appeared in the article’s title. Most of the studies (51%) were of a general theoretical kind and only 31% dealt with issues related to gender. Out of the 45 articles on ethics of care only 17% were based on empirical research within the field of psychology.

As these alternative theories have developed over the last 15 years, the influence of ethics of care has waned. However, the sheer indifference of contemporary moral psychology to issues of care, relations, and dependency, suggests that other reasons beyond the emergence of alternative theoretical models may have been responsible for the diminished influence of ethics of care in contemporary moral psychology.

The next segment will examine the possible reasons for the theory’s irrelevancy to moral psychology, and draw a number of conclusions that may be helpful to our current understanding of this discipline.

## REASONS FOR ETHIC OF CARE’S IRRELEVANCY TO MORAL PSYCHOLOGY

The first reason for the irrelevancy of care ethics to moral psychology is the name of the theory itself: the title places an emphasis on the philosophical rather than the psychological application of the theory. Thus, the latter merely serves as a backdrop to the ethical aspects but is not researched in its own right.

The reason why ethics of care triumphed over the psychology of care is directly related to the debate between Kohlberg and Gilligan. Kohlberg (1963a,b), was deeply influenced by Kant’s moral theories. His six stages of moral development are in line with Kantian moral perceptions.

Thus, a subject reaching the most advanced stage, known as universal ethical principles, would know that laws are valid only insofar as they are grounded in justice, and that a commitment to justice carries with it an obligation to disobey unjust laws. Kohlberg would consistently espouse a rationalist view in which reason takes account of emotion. However, reason, not affect, was always for him the ultimate judge.

Gilligan, on the other hand, offered a different view on ethical reasoning. While investigating the moral thinking of women, she found that mature, adult, intelligent women, do not think about moral problems in an abstract and impersonal way or in terms of justice, but rather in terms of personal moral responsibility. Responsibility for and protection of the other, Gilligan suggested, is at the core of women’s moral thinking. Men, on the other hand, see a moral problem as one that can be resolved in terms of right and wrong. Gilligan argued that although the reasoning of women when making moral choices is different, it is no less logical or mature than that of Kohlberg and Kant.

The argument, therefore, is not only a question of the existence or nature of gender differences regarding patterns of moral thinking. Rather it is a debate about what form of moral thinking is more developed, mature or advanced – in this case the logical versus the compassionate. Thus the argument steered away from psychology and became part of the realm of philosophy.

The second reason for the irrelevancy of ethics of care is linked to the fact that the theory showed little interest in the questions that preoccupy moral psychology such as; what is the basis of morality (cognition, intuition, or culture)? What are the sources of evil? How is one supposed to draw up the parameters of blame ascription?

In fact, ethics of care took a closer interest in the psychology of women than it did in moral judgment. The idea that women define themselves through a web of relationships of intimacy and care rather than through a hierarchy based on separation and self-fulfillment, runs as a leitmotif through Gilligan’s theory giving it much of its structure and attraction.

The developmental theory espoused by ethics of care is also not exactly a theory of moral development. Indeed, it should rather be viewed as a theory of the development of gender identification, achieved through the child’s relations with its mother. Whilst girls attain their gender roles through resemblance and connectedness with their mothers, boys attain them through distinction and separation. Therefore, in adolescence, boys have to learn how to deal with relationships *despite* their fundamental sense of separateness and distinctiveness, whereas girls must grapple with the task of establishing their distinct identity while holding on to their relationships with others.

Ultimately, Gilligan (1982, p. 100) argues, men and women claim different moral imperatives: women feel “a responsibility to discern and alleviate the ‘real and recognizable trouble’ of this world,” whereas men’s moral imperative “appears rather as an injunction to respect the rights of others.” From this we can see that moral judgment is a product of the different patterns that characterize men and women.

The third reason for the irrelevancy of care ethics to moral psychology is linked to its close tie with feminist ideology. This tie was important in the past because at the time moral psychology served as a means of excluding and derogating women. For example, Piaget (1965/1932, p. 77) wrote that “The most superficial observation is sufficient to show that, in the main, the legal sense is far less developed in little girls than in boys.” Kohlberg’s moral development scale paved the way for similar thinking, providing biased measures for those who wish to show that, once again in this area too, males are superior.

Gilligan’s contention is that the lack of female development assumed in such studies was the outcome of an injustice inherent in the research itself, which, she claimed, was essentially based on research into male subjects. The result, according to Gilligan (1982, p. 6), was that the observations of psychologists were biased; by “implicitly adopting the male life as the norm, they have tried to fashion women out of a masculine cloth.” The specific moral development of women, Gilligan (1982, p. 31) noted, “falls through the sieve” of a male dominated tradition of research.

In fact, Gilligan found that the voices of women had either been excluded from the research on ethics or had been considered inferior. She thus embarked on giving expression to the voice that had not been heard.

As critics pointed out, the problem was that this single-minded focus on women’s own culture, risks ignoring the larger social and historical developments of which it was a part (Kerber, 1986), hence maintaining cultural exclusion. As a result, it trapped the important ideas linked to ethics of care in an isolated ideological ghetto where everything entering that ghetto was automatically identified with the idea that women were different from, and usually superior to, men (Dubois et al., 1980).

The fourth reason for the irrelevancy of ethics of care is linked to the difficulty that the theory had in substantiating its assumptions through empirical research. In Walker (1984) published a broadly based study which analyzed 61 works of research using Kohlberg’s model to assess the level of moral reasoning achieved by both genders. This showed that, in general, there were no differences between the sexes either in childhood or adulthood. Moreover, it demonstrated that in those instances where such disparities were identified, the data included in the study revealed that the men in question were more highly educated than the women surveyed. Therefore, education, not gender, was the explanation for the women appearing to have achieved a lesser level of moral reasoning. There is no indication in Walker’s comprehensive study that the two sexes follow separate paths in relation to their moral thinking about conceptual, theoretical issues.

Gilligan (1986), disagreed with the results of this research, insisting that other studies had, in fact, found differences in moral thinking between men and women. Specifically, Gilligan claimed, there were findings showing that men tend to define and resolve moral problems within the framework of the justice system, even though they do admittedly introduce considerations of care into their thinking. Girls and women, on the other hand, focus on care in moral reasoning, making the concentration on care a typically female phenomenon.

The fifth reason for the irrelevancy of care ethics, is linked to the fact that the debate between Kohlberg and Gilligan was mainly about peoples’ reasoning in relation to moral situations. This debate became very outdated when moral psychologists found that reasoning played a negligible role in moral judgment. What this finding in fact claimed, was that much like other cognitive processes moral judgments appear in consciousness automatically and effortlessly as the result of moral intuitions and before any conscious processing has occurred (Bargh and Chartrand, 1999). Moral reasoning, it follows, is an effortful process in which a person searches for arguments that will support a judgment already reached (Haidt, 2012). Emotions and affects are the basis of judgment and not logical arguments (Haidt, 2000).

Again, ethics of care had little to say on these matters. In fact, the debate between Kohlberg and Gilligan was so central to the identity of care ethics that when Kohlberg’s theory began to be outdated and lose its relevance the same happened to ethics of care.

## REPLACING ETHICS OF CARE WITH PSYCHOLOGY OF CARE

If we were to strip the psychology of care ethics (elsewhere I have called it an attachment approach to moral judgment, Govrin, 2014), of all the components that have been refuted by empirical research, we would be left with just one main psychological element of the theory: the idea that care giving and the primary relations between mother and child are of central importance to moral judgment. This idea constitutes a breakthrough in moral psychology that is yet to be properly understood. Though originally ethics of care emphasized this link, it did not dwell on its universal importance to the development of moral thinking for both men and women.

As opposed to ethics of care, psychology of care should base its development on our common universal experience of caring and being cared for as a child. Human survival is dependent on the existence of caring relationships, a fact that clearly applies to both genders. Infants would not survive were it not for the presence of a caregiver. Moreover, the practice of care is also of importance when it meets more than just the bare requirements of survival. Having survived infancy, children are unlikely to develop well in the next stages of life unless they are loved and valued for who they are. By stressing the extent to which humans are dependent and in need of significant levels of care from others, care psychology can make a hugely important contribution to moral psychology. Any moral psychology that does not take dependency into account is necessarily inadequate (Held, 2006).

The idea that the care given at the beginning of life is central to the development of moral mechanisms, is consistent with moral psychology’s search in recent years for the “universal factor” underlying moral judgment. For example, an important theory in moral psychology known as Universal Moral Grammar (UMG), is ultimately predicated on a belief that every human being possesses a faculty of moral judgment, the normal development of which is largely unaffected by racial, cultural, or even educational differences (Mikhail, 2007). A view commonly expressed today is that we are born with certain abstract rules or principles which set the parameters and guide us toward the acquisition of particular moral systems. Researchers who adopt this view often compare moral acquisition to language acquisition (e.g., Hauser, 2006; Roedder and Harman, 2010).

If, indeed, such a comparison is valid, it follows that, similarly to what happens with language acquisition, there has to be a stimulus that triggers the acquisition of morality. Care is the appropriate stimulus that is likely to lead to the learning of the proper processes which guide us in the making of moral judgments.

As we shall see, infant research shows that meaningful social learning takes place through the interaction between the infant and the caregiver. If psychology of care was able to demonstrate the way in which such learning are linked to morality, it would have the potential to show the following: that the universal foundation of our moral faculties is linked to the fact that all humans are born into a state of absolute dependence on the mothers who raise them. And, secondly, that the learning which grows out of this context enables us to distinguish between right and wrong.

In order to substantiate the logic of this thesis I will attempt to establish the idea that the learning that occurs in the first year of life creates the infrastructure and the basis for learning at a later stage. In addition I will argue that this is linked to the initial interactions between the infant and the caregiver and can lead to the development of moral faculties (For a detailed model see Govrin, 2014).

## JOHN BOWLBY AND THE ORIGINS OF MORALITY

More than 60 years ago, the British psychoanalyst John Bowlby developed a highly innovative view of the process by which infants acquire moral sense through their attachment to their caregivers. Much like the originators of ethics of care, Bowlby believed that the key role in this process is the intra-psychic structure and affective experience that are developed in the infant–caregiver bond.

Most people are familiar with attachment theory’s division of attachment into four styles. However, a more fundamental subject of research is how the initial attachment shapes the organization of our thinking and determines our emotional syntax (Fonagy and Target, 2007). Bowlby linked delinquency to a breach in the relationship between the child and his mother. In his study of 44 juvenile thieves (Bowlby, 1944), he explored the link between the nature and extent of the child’s disorder and resultant delinquent behavior, as well as the question of when, and in what way, the breach between mother and child first occurred (Bowlby, 1958, 1969).

In Bowlby’s view, when it comes to attachment issues, the critical period in the infant’s life is between the ages of 6 months and 3 years. He believed that deprivation at any time after the first 6 months of life was likely to seriously affect the child’s ability to try and become emotionally involved with other people; to love, to trust, or to feel safe in having and giving expression to conflicting emotions. This was due to the fact that experiencing separation at that particular stage of life would, in all probability, interfere with the still emerging realization of his dependence on others. Today we know that the etiology of delinquency is far more complex than Bowlby thought, and is influenced by many factors. However, Bowlby paved the way for the establishment of attachment as the foundation of moral development.

Ethics of care and attachment theory have essentially the same view about attachment as a key factor in moral behavior. Both share the idea that the objective quality of parental care is central not only to the child’s survival but to his experience of himself and of the world. Thus, the two theories complement each other. Attachment theory after Bowlby paid little attention to moral development and when it did so it was in general terms. Ethics of care included an expansive reference to moral psychology but paid little attention to the care given during the first year of life in which the processes of gender identification have still not begun. Combining the two theories would help to establish a theoretical psychological infrastructure for psychology of care that will provide us with a more comprehensive explanation of contemporary findings.

## THE FORGOTTEN MOTHER- THE CAREGIVER ROLE IN THE DEVELOPMENT OF MORAL JUDGMENT

Bowlby’s attachment theory was the first psychological proposition to place maternal care at the center of human psychological development. But even 60 years later, when researches are exceedingly aware of the fact that moral and social faculties already begin to develop in the first year of life, they do not make room for, or place any importance on, the maternal role in this development. In most of the studies, infants are still being perceived apart from their surroundings as if they possessed an isolated mind that was developing separately from the environment in which they were growing up. For example, Hamlin et al. (2010), argue that the capacity of infants to evaluate individuals on the basis of their social interactions is unlearned. Likewise, Hoffman (2001), one of the most prolific writers in the field, thinks that the empathic and social faculties of infants develop “naturally” without prior experience.

Even though infants’ social and empathic abilities are a salient feature of any contemporary book on moral development, the role of the caregiver in moral development was not directly theorized nor studied. This lacuna has a long history in moral psychology that stretches from Piaget and Kohlberg until the emergence of care ethics and now within contemporary moral psychology.

Why is the centrality of the infant–caregiver bond so easy to dismiss? As Mitchell (2000) points out, this tendency to ignore and dismiss must surely have something to do with confusion about the way in which the development of the mind differs from that of the body. Our body’s sequence of development seems to be more or less pre-programmed. This manifests itself in the way in which, with the passage of time, people mature in an ordered way. From immobility, the infant progresses to being able to turn over, then to pull himself up, then to crawl and finally to walk. Aside from those who suffer from a severe physical handicap of some sort, we all ultimately gain control over our bodies and become physically functional almost entirely through our own efforts. It is tempting to believe that our minds develop in a similar way. This temptation leads us to take for granted our independent mental existence in much the same way as we presume our independent physical existence.

The next segment will summarize some of the important research accumulated on infant development during the first year of life. This will aim to show that the probable centrality of the caregiver during that early period of life has a great influence on the moral representations the infant acquires.

## THE IMPORTANCE OF THE FIRST YEAR TO THE DEVELOPMENT OF SOCIAL AND MORAL JUDGMENT

Over the past 20 years, as infant research has become increasingly more sophisticated and complex, we have learned that infants possess a much more intricate and far richer knowledge of the world than had previously been supposed (Mandler and McDonough, 1998). This knowledge precedes the development of gender consciousness.

Infants learn a great deal about their physical world (e.g., Gelman, 1990; Spelke et al., 1992; Baillargeon, 1993), and the knowledge they accumulate during the first year of life forms the foundation on which later learning, including language acquisition, counting, object categorization, social relations, and other complex cognitive skills, rest (e.g., Wynn, 1990; Mandler and McDonough, 1998).

As far as morality goes, we now know that at 7 or 8 months infants have specific capacities related to moral judgment (e.g., Meltzoff and Moore, 1995; Gergely, 2011), which enable them to judge a person’s character by his behavior toward others. For example, at 8 months infants show a preference for a character that is actively helpful to others as opposed to someone who is indifferent to those around him. They have even less of a preference for a character that actively hampers the progress of others. This faculty may be the basis of moral thought and action in later life (Hamlin et al., 2010).

Similarly, there is a great deal of evidence to suggest that expectations of social relations emerge in the first months of life through infant–caregiver interactions (for reviews see Beebe, 2005). For example, the infant develops an awareness of a principle known as “ongoing regulations” (Beebe and Lachmann, 2002, p. 60), which enables him to form and organize basic representations of mother-infant interactions which will later in life help him to predict certain behaviors and their consequences. If his expectations are violated, he will invest a great deal of effort in resolving these breeches, a principle known as “disruption and repair.” Consistently, infants were found to be affectively reactive to violations and confirmations of anticipation (DeCasper and Carstens, 1980). It is from these primary interactions that the infant develops expectations of relational patterns, remembers them, and categorizes them (Hauser, 2006).

Thus, the research showed that infants learn the basic principles of relations between two people. They develop a whole array of expectations by observing their caregiver. There are reasonable grounds for assuming that later on in life moral judgments are made on the basis of these expectations. Infants respond with unease to any violation of an expectation.

Therefore, a psychology of care that supersedes ethics of care can show how the initial interactions of infants with their caregiver prepare them for the acquisition of knowledge that later on will be relevant in conceptualizing moral situations and the creation of the in-depth structures of moral knowledge.

### MORAL JUDGMENTS IN TERMS OF EXPECTANCIES AND TRANSGRESSIONS

One can regard moral failure as a violation of our expectations. When an individual acts contrary to our expectations of him we usually consider their action wrong. When individuals act in a way that is consistent with expectations, we assume their action to be right, even if we do not openly classify it as such (Hauser, 2006).

As said, the array of an infant’s expectations develops out of the initial contact with the caregiver. If care represents the expectations the infant has of the caregiver – the feelings, actions, thoughts that are intended to protect the infant and raise him – then moral failure represents the very opposite. The infant can develop expectations of the caregiver – an adult who is powerful and independent, will meet his needs, and offer protection. From this comes the idea that the strong and the big have to take care of the dependent and the weak. It may be that this becomes part of intuitive knowledge acquired outside of consciousness and the means by which we get to know how to analyze a conflict between two sides. On the basis of the infant’s first experiences, an ability develops to identify in every conflict which of the two sides is strong, responsible, mature, and in command of resources, and which side is dependent and helpless. Thus, in an intuitive way, defined and clear expectations of the strong side develop that relate to what he should and should not do to the weak and vulnerable side. When we decide that someone is guilty of a moral transgression, we can see it as a violation of the expectation of the way the strong and independent should behave toward the weak and dependent. In this view, judging moral situations means finding the asymmetry between two parties. This might be the deep structure of all moral harm, the similar common characteristic. It may be that breaking a conflict down to its constituent parts and finding the “strong” “adult” and the “vulnerable dependent” is part of an innately set faculty. The child is somehow prepared, ready, and able to acquire this capacity by using experience from his first interactions with the caregiver. This experience is used to acquire the “core” syntax of moral judgment.

This thesis can be enhanced by a series of studies by Gray et al. (2012). Gray showed that moral judgments do not depend merely on the superficial properties of moral events, but also on how those events are mentally represented.

One of Gray’s most important findings is that moral judgment is rooted in a cognitive template of two perceived minds—a moral dyad of an intentional agent and a suffering moral patient. Intentional agents are capable of intent and action due to capacities for judgment or self-control, whereas moral patients are ones who are capable of experiencing physical and emotional sensations.

Adult humans usually possess both characteristics of patients and agents and can therefore be both blamed for evil and suffer from it. A puppy, according to Gray, is a mere moral patient: we seek to protect him from harm but do not blame him for injustice.

Gray posits that despite the variety of moral transgressions, the moral dyad not only integrates across various moral transgressions but also serves as a working model for understanding the moral world. Through the dyadic template of morality we typecast people into two categories – moral agents or moral patients—a phenomenon Gray called moral typecasting. Typecasting determines our perception of the target person’s mind. A person who does something evil will be immediately categorized as a moral agent. This means that simply doing something good or evil can bring with it corresponding attributions of intention, especially evil intentions. Likewise, when someone is categorized as a moral patient, people automatically infer the capacity for experience and greater sensitivity to pain (Gray and Wegner, 2009).

Psychology of care can therefore show that the moral dyad is formed in our mind as a result of our inner schemas of children (moral patients) and adults (moral agents) that we acquired in the first year of life. Thus, the rapid intuitive conceptualization between moral patient and moral agent has its origins in the period of care in which there were asymmetrical relations between the two sides and in which the caregiver had to tend to the infant’s needs. These interactions developed the infant’s expectations about relations.

Gray thinks that agency is the factor that can distinguish between the two sides of the dyad. If so, it makes dependency the central feature of the dyad. Adult-like or child-like dimensions are not necessarily related to specific age but to the quality of a person or interaction. To put it more accurately, when we make a moral judgment we are looking for cues of dependency and independency. For example, people unconsciously associate disability with child-like features (Robey et al., 2006). In another study, college students addressed people they believed to be adults with disabilities much as they were in the habit of addressing a 12-year old child (Liesener and Mills, 1999). The “detection” of child-like and adult-like characteristics is not entirely rational and not always relevant. For example, a number of experiments (e.g., Berry and Zebrowitz-McArthur, 1985) indicate that baby-faced people are less likely to lose their case than people considered to have an “adult face” (Berry and Zebrowitz-McArthur, 1988; Zebrowitz and McDonald, 1991). If found guilty, a baby-faced defendant will be considered less likely to have committed an offense intentionally, and more likely to have offended by being negligent than would a defendant with a mature face.

What might be further added to Gray’s account is the element of **expectations from the caregiver**. Even if we match each party to moral patient and moral agent schemas as Gray suggests, the judgment remains incomplete. We do not simply compare the two parties individually and decide which one is more helpless, needier, or more powerful. Our judgment depends on something much more profound. It is linked to the nature of the dyadic relations. Just as we have different schemas for adults and children, so we have a schema for the dyadic relations between them.

This representation consists of our expectations of what adults should and should not do to children. Adults have obligations toward children and we seem to know these obligations by heart. Moral transgression might be perceived as violating our expectations of moral agents to act in certain ways toward moral patients. Exploring mother-infant interaction in the first year of life teaches us how the infant’s expectations of the patterns of behavior of moral agents toward moral patients is formed and how the infant develops pre-symbolic representations of moral dyads. Psychology of care can lift the collaboration between moral psychology and infant research to an exciting and creative new level.

## MORAL JUDGMENT AND THE HUMAN VISUAL SYSTEM

If humans treated every new right–wrong situation as a novel and unique experience we would quickly drown, our minds baﬄed and confused. Conceivably, to make the judgment more efficient, the cognitive system groups moral situations into the meaningful category of the Adult – Child format. Various aspects of moral situations are perceived holistically rather than separately or independently.

Gray et al. (2012), suggest that if our template of morality were dyadic – perceived intentional moral agent and a suffering moral patient – we would be compelled to complete the moral dyad if it appeared to be incomplete. For example, when we see someone blameworthy—an apparent moral agent—we would complete the dyad by inferring the presence of another suffering mind —a moral patient. Gray suggests the phenomenon of dyadic completion occurs at an intuitive level—like the Gestalt completion.

We do not know what neurobiological framework accounts for the completion of the dyad. Most cognitive psychological moral theories are formal and detached from neuroscience. I suggest that much can be gained by taking advantage of the large amount of information available on the neurophysiology of visual recognition. Although moral judgments and visual recognition are separate, unrelated domains, what might be of interest to us is the ability of the brain to fill in missing elements so that visual recognition remains largely unaffected by the absence of such components. Basically the thought is that visual images constructed by the brain are holistic- i.e., are more complete than one would expect from the linear sum of their individual parts. Human brain imaging research has strongly supported such thinking by showing that one cannot explain the neuronal activity measured in high order visual areas in response to a picture as representing the sum of the responses to the picture’s elements.

Although visual recognition is a perceptual phenomenon, it can also be regarded as an ubiquitous property of various types of neural network models (Williams and Jacobs, 1997; Ullman, 1998). Such networks, upon being presented with a partial input pattern, can settle quite rapidly into an attractor state matching the complete stored pattern (Lerner et al., 2002).

Studies point to the lateral occipital complex (LOC) as a central site in which object completion effects are manifested. Other studies show that infants only a few months old complete representations of objects even behind occluders (Kellman and Spelke, 1983). Psychophysical experiments on adults suggest that such completed representations determine the allocation of visual attention (He and Nakayama, 1992).

One could argue that in the same way that areas in the brain play a critical role in object completion other areas are dominant in the completion of the dyadic Gestalt.

## “HEINZ’S DILEMMA” AS SEEN FROM THE PERSPECTIVE OF A PSYCHOLOGY OF CARE

Gilligan attempted to refute the claim that the moral reasoning of women is immature because of its concern with immediate relations. The “care perspective,” Gilligan asserted, was an alternative and equally valid form of moral reasoning unnoticed by masculine liberal justice traditions which, she argued, are driven by notions of autonomy and independence.

Gilligan expressed these thematic perspectives through the moral reasoning of “Jake” and “Amy,” two children in Kohlberg’s studies responding to the “Heinz dilemma.” In this dilemma, the children are asked whether “Heinz” was justified in stealing an expensive drug to save the life of his sick wife. Jake perceives the Heinz dilemma as a mathematical problem. Seen from this viewpoint the right to life wins over the right to property, so that all “reasonable people” should conclude that Heinz was justified in stealing the medicine. Amy, on the other hand, disagrees with the idea that Heinz’s theft was justified. Her concern is that he might be sent to prison and that his sick wife would consequently be left on her own. For Amy, the dilemma is a narrative of relations over time involving ruptured links which have to be repaired through interaction. Amy’s understanding of the world is that its inhabitants are not isolated from each other but rather belong to networks of relationships. She is confident that once the pharmacist realizes why Heinz stole the medicine he would be willing to cooperate with Heinz.

Gilligan posited that men and women often speak different languages which they think are the same. She used this idea to try and moderate moral psychologists’ tendency to adopt the “male perspective” as the model of good moral reasoning.

From the perspective of contemporary moral psychology, Gilligan erred in attributing a great deal of importance to the arguments underlying moral judgment. First of all, such arguments are retrospective and are voiced only after the judgment has already been made (Haidt, 2000). They therefore play a negligible role in the decision making process. Secondly, as said, research has revealed that, contrary to Gilligan’s view, arguments based on “justice” and “compassion” are used by men and women equally. It is important to point out that though their reasoning differed, Amy and Jake came to an identical conclusion regarding the importance of saving Heinz’s wife. From the viewpoint of a psychology of care, the most important factor is that all those who were surveyed, other than children less than four, regarded Heinz’s wife as a severely ill woman who had to be saved, even if that meant undermining another individual’s property rights. Even though they used different arguments they gave the “right” answer. From this perspective, Heinz’s dilemma can perhaps be seen as constituting a challenge to the capacity of moral reasoning, but does not represent a serious moral dilemma. According to the psychology of care, in order for Heinz’s dilemma to become a real moral issue in which the participant has to decide between different moral choices (as opposed to various arguments related to the same choice) the “attachment” between the participant and Heinz’s wife or Heinz himself has to be challenged. This can be done in one of two ways: by reducing the elements of dependence/neediness attributed to the wife, or by stressing the “dependency” – in this case the child-like features of the chemist (or his family) from whom Heinz stole the medicine. If, for example, Heinz’s wife is revealed as a woman who committed a series of brutal murders in her past, there are reasonable grounds to assume that that the participants would hesitate before deciding whether the theft of the medicine was justified. Similarly, they would be highly tentative about reaching a conclusion if they were to discover that as a result of the theft the chemist had lost an important source of income intended for a heart transplant operation urgently required by his infant son. This restructuring of the experiment turns the Heinz conundrum into a real moral dilemma. In that they weaken the understanding of Heinz’s wife or re-enforce the concern about the chemist they are liable to make it difficult for the brain to think quickly and intuitively in Heinz’s favor and reach a clear cut decision. And indeed what is common to all serious moral dilemmas (such as aggressive methods of questioning terrorists or the morality of the Allied bombing of Dresden during World War II) is the difficulty of reaching an unequivocal decision as to the extent of the neediness/dependency/weakness of the victim and the aggressor.

## PHILOSOPHICAL IMPLICATIONS AND THE IMPORTANCE OF MORAL LAWS

The philosophical implications of the psychology of care are significant and cannot be detailed here. I will, however, dwell briefly on one issue.

Ever since the days of Aristotle and Plato there have been disagreements in moral philosophy between “ rationalists” and “intuitionists” about the true nature of moral judgment (Beauchamp, 2001). The rationalists posit that people reach a moral decision by thinking about the rights and wrongs of each case and then making a deliberate and conscious moral judgment (e.g., Kant, 1785). Intuitionists and sentimentalists on the other hand, claim that people reach moral judgments instinctively and can make such judgments non-consciously (e.g., Hume, 1739).

To a large extent the drive behind care ethics is the belief that the theories of the rationalist mainstream – and in particular Kantian ethics, utilitarianism and liberalism – provide an insufficient basis for the making of moral judgments. All three approaches, it is argued, overlook the part played by people’s emotional responses – especially empathy, sensitivity, and their reaction to particular “others” – in reaching moral decisions. Ethics of care further argues that utilitarianism and Kantian ethics reduce moral understanding to the presentation of a single principle and consider “abstract rules” to be the foundation of moral guidance.

Psychology of care can be a link between these two approaches. Whilst the protection of the young is very common among mammals, humans, by virtue of their developed abilities (symbolic, linguistic, and logo- mathematical skills), are able to forge out of what are essentially biologically driven patterns of behavior a set of general and abstract principles. For example, the features associated with an infant such as neediness, dependency, and helplessness, are part of virtually every culture. They have also been extended to additional populations such as the elderly and the handicapped because the neediness and injury of these communities can be likened to the dependence and injury of infants/children. Feelings we have toward children are directed at a number of populations identified by their particular characteristics. Moral feelings are a combination of cognitive and emotional abilities. The cognitive achievement is the ability to equate those who belong to the moral community with dependents. It includes the generalization of feelings to one’s own child and to other individuals on the basis of similarity (i.e., Handicapped = child). The emotional capacity directs the array of feelings such as concern, compassion, and sympathy – originally focused on the person’s own progeny – toward other unrelated needy individuals. Secondly, humans have the ability to enact abstract laws related to moral situations. They can, for example, imagine moral situations prior to them happening or think about various values behind moral situations (“the sanctity of life” “the right to freedom”). Humans have the capacity to enact laws ordering the relations between people which determine norms of behavior. They do not need specific dyads in order to understand dyadic laws and in order to formulate them. Basic laws and moral principles play a very important role in society.

Without laws to guide him, the individual psychological mechanism which is part of every person’s make-up cannot be a good basis for a moral judgment to be made. Though the mechanism of breaking down the moral situation into its constituent parts of caregiver/infant is universal, the substantive decision is culture dependent and subjective. The vastly different ways in which people react to moral issues stems from the fact that the judgment concerning which dyads “activate” the moral mechanism and which dyads do not, is an entirely subjective decision. Not every asymmetrical moral situation, or set of circumstances in which the weak party is hurt, is perceived as a moral violation. There are many situations in which ostensibly the “strong party” has hurt the “weak party”. However, the situation in its entirety does not activate the affective and cognitive mechanism required in order to reach a moral judgment. The psychic system, especially its affective parts, simply does not interpret the situation as “strong hurts weak.” This may be due to a number of reasons. For example, it may be that the “weak” party has been “dehumanized” to the extent that the empathic response to its injury is muted. There may be an identification with the “strong party,” or an understanding of the reasons which have led it to harm the weak. Alternatively, it may be that the “weak party” is thought to be to blame for what has happened or is perceived as dangerous. The personal values which each one of us believes in also enter into this mix. The instability and caprice of the subjective moral system is not a mechanism one can rely on in maintaining a moral and lawful society.

General moral principles such as those suggested by Rawls and Kant, enable us to rise above the subjective dyad. Turning the moral mechanism into a universal law enables us to protect the weak and prevent moral injustices, independently of the emotional mechanism that links an individual to a particular dyad. In the absence of this human ability to establish abstract laws there would likely be an anarchic situation in which the moral mechanism as described would be activated arbitrarily in line with the individual interests and needs of every single person.

The ability to enact a moral law is a human achievement of the utmost importance. It enables society to dictate to people who should be protected – in other words who is within the moral community and is therefore worthy of protection – instead of leaving this judgment in the hands of anyone and everyone. Laws such as Kant’s categorical imperative or Rawls’s veil of ignorance are aimed at conceptualizing the psychological mechanism to such a degree that the moral decision is distanced from the concrete dyad and subjective feelings and is determined solely by noble values of justice and morality.

It is important to understand that Kant and Rawls moral principles are consistent with the psychological mechanism presented here. They merely introduce additional cognitions which had not previously been taken into account. Whereas the “natural” psychological mechanism identifies people as child-like on the basis of resemblance and membership of that same group, the moral principles compel us to ignore this component and relate to all suffering people as child-like. In other words, the moral principles employ the same parameters to moral judgment; a dyadic structure drawing a dividing line between child-like and adult-like, “computing” the relative strengths between the two parties, and assessing the extent to which expectations have been violated. However, in the course of this assessment the moral principles require us to entirely ignore our affinity to one or other of the two parties in the dyad on the basis of resemblance, shared interests, or any other kind of subjective factors besides the parameters of the general dyadic rules.

Let us assume that an individual experiences a sense of identity with and empathy for members of his own national group but that he has no such feelings for other nationalities. However, the moral principle dictates that every person is entitled to a set of basic human rights irrespective of which national grouping he or she belongs to. This cognition makes moral judgment far more complex. From then on, when making a moral judgment the system has another constraint: it will hesitate before allowing itself to offer any form of discriminatory preferential treatment to people belonging to that particular national group. It will direct itself to recognize the suffering of anyone who is not a member of that nationality. Though the affective mechanism can detach itself from the new constraint, as indeed often happens, it is nonetheless capable of including this cognition in reaching its judgment. Moral principles such as those of Kant and Rawls have an interest in extending the mechanism of the psychological system to apply to all peoples on the basis of equality. They teach us to curb personal considerations and subjective associations and be guided by our natural system to reach a moral judgment by relying solely on one consideration: to what extent did the adult-like party (any human being) violate our expectations by the way in which he behaved toward the child-like party (any human being) in any given moral dyad.

## CONCLUSION

Ethics of care was the first theory to challenge the Kohlbergian – Kantian view that moral judgment is determined by rational psychological processes. In moral psychology it was the first theory to present a model of moral judgment based on emotions. The empirically based research findings in moral psychology consistently indicate that ethic of care’s intuition in the matter was correct: emotions do indeed play a decisive role in moral judgments. However, the theory itself has become marginalized and irrelevant. Worse still, although contemporary infant research has demonstrated that infants possess basic moral faculties, the caregiver’s role in the development of these faculties and the influence of parental care on the newborn has been entirely abandoned. Only in feminist or psychoanalytic theories are interactions with the infant regarded as central to moral development.

Ethics of care uncovered an important and universal axiom of human ethics. Throughout the history of western thought language, morality, and the sharp division between reason and emotion, have been employed to exclude women (and other groups outside the white male dominated mainstream) from being acknowledged as rightful contributors to knowledge.

In this matter, care ethics have played a significant role by focusing on the importance of affect and emotion to reassessments of rationalism and the assumed role of impartiality in the accumulation of knowledge. As Greeno and Maccoby (1986, p. 310) have noted: “Gilligan’s book was intended to right a wrong.”

But today, empirical studies show that infant–parent interaction seems to be an adequate moral imperative for all men and all women and the association of parental care with women’s morality alone is less relevant.

If psychology of care is to succeed in the long run as a moral psychology, it must be bolder and more revolutionary, reshaping the core of moral psychology. If psychology of care is to flourish in the 21st century, the prevailing theoretical frameworks must be discarded and replaced with a single integrative model that seamlessly connects with cutting-edge research in mainstream psychology. A new paradigm for psychology of care, an attachment approach to moral judgment, must emerge or the theory will perish as a moral psychology. I suggest that an advance in our understanding of the way care in the first year of life organizes the mind, is an opportunity to create closer ties between the previously separate domains of moral psychology, ethics of care, infant research, and attachment theory.

## Conflict of Interest Statement

The author declares that the research was conducted in the absence of any commercial or financial relationships that could be construed as a potential conflict of interest.
